# Kidney Transplantation in Congenital Abnormalities of Kidney and Urinary Tract (CAKUT)

**DOI:** 10.3390/biomedicines13040932

**Published:** 2025-04-10

**Authors:** Silvio Maringhini, Lars Pape

**Affiliations:** 1 Department of Pediatrics, IRCCS-ISMETT (Istituto Mediterraneo per i Trapianti e Terapie ad Alta Specializzazione), Via Ernesto Tricomi, 5, 90127 Palermo, Italy; 2 Department of Pediatrics II, University Hospital of Essen, University of Duisburg-Essen, Hufelandstraße 55, 45147 Essen, Germany; lars.pape@uk-essen.de

**Keywords:** congenital abnormalities of the kidney and urinary tract (CAKUT), chronic kidney disease (CKD), kidney transplantation (KTx)

## Abstract

Congenital anomalies of the kidney and urinary tract (CAKUT) are a common cause of chronic kidney disease in children. Most patients will reach end-stage renal function and dialysis or transplantation in childhood or early adulthood. Patients with CAKUT deserve a careful evaluation before a kidney transplant; detailed imaging and functional studies are necessary, particularly in the presence of lower urinary tract abnormalities, and surgical procedures are advisable in selected cases. A higher incidence of complications has been reported after a kidney transplant in CAKUT, mainly urinary tract infections. However, in the long term, the prognosis seems to be comparable to other kidney diseases. A large number of reports are available in the literature on medical and surgical management of patients with CAKUT before, during, and after a kidney transplant; almost all recommendations of surgical procedures before a kidney transplantation are based on retrospective not controlled studies or personal opinions; prospective controlled studies are needed. In this narrative, nonsystematic review, we report the results of recently published selected studies and underline questions that should be addressed in future guidelines.

## 1. Introduction

Congenital anomalies of the kidney and urinary tract (CAKUT) include a large group of kidney diseases primarily affecting children who then enter adulthood. CAKUT may be classified depending on the sites involved in the urinary system: kidneys, upper urinary tract, and lower urinary tract. In many cases, different parts of the system are involved together [Table 1] [1]. CAKUT can be familial, sporadic, and syndromic. Genetic causes can be demonstrated in most syndromic CAKUT cases; in sporadic and familiar CAKUT, a large number of cases recognize a monogenic cause (54 genes have already been identified) or have pathogenic copy number variants [2,3,4,5]. CAKUT represent the most relevant cause of chronic kidney disease (CKD) and end-stage renal disease in children (ESRD) in Western countries [6]. Kidney transplantation is the therapy of choice for all children with ESRD; however, a pre-transplant evaluation with a careful urologic assessment is necessary in most cases of CAKUT to secure long-term graft function [7]. Graft survival in children with CAKUT is comparable with that of patients affected by other kidney diseases [8]. Several reports and reviews are available on kidney transplantation in CAKUT, but no guideline has been published until now. This paper is a narrative review; we started with a PubMed search of original papers and reviews dealing with the various aspects of kidney transplantation in CAKUT covered in our paper and extracted from selected papers additional references that were not retrieved in the original search; we will underline unsolved questions. Even though this is not a systematic review, ours is an orderly, well-organized approach.

## 2. Epidemiology

The prevalence of CAKUT has been reported from 4 to 60 per 10,000 births [1,9]. The most frequent congenital malformation is urinary tract dilation that regresses spontaneously in the large majority of cases and does not lead to terminal renal failure. Renal dysplasias, hypoplasias, agenesis, horseshoe kidneys, obstructions at the ureteropelvic junction or the vesicoureteral junction, posterior urethral valves (PUV), and vesicoureteral reflux (VUR) are common variants of CAKUT and may be associated with other diseases (syndromic CAKUT) [10]. Genetic, epigenetic, and environmental factors contribute to the development of CAKUT. Several genes have been identified as responsible for CAKUT: single gene mutations account for 10–20% of sporadic cases, and 4–15% are associated with pathogenic copy number variants [5,11,12,13]. In the future, larger cooperative genetic studies will probably allow for the identification of causative genes in a larger percentage of cases. Neurogenic bladder (NGB) is a condition in which the bladder dysfunction is a consequence of congenital or acquired neurological conditions affecting the innervation of the lower urinary tract. Patients affected by CAKUT may have mild or no symptoms of bladder dysfunction during their childhood, and adult nephrologists often look after them since, if not treated in childhood, they often develop evident problems in adulthood [14]. Dysfunctional voiding is often neglected in patients who receive a kidney transplant, which may be jeopardized by complications of this condition. Almost one-third of adults on renal replacement therapy (RRT) have no definite diagnosis of their renal disease; slowly progressive CAKUT may be the cause for a substantial number of these patients; more investigations should be carried out on patients enrolled for a kidney transplantation.

## 3. Pathogenesis of CAKUT and Kidney Damage

Several factors may be implicated in the pathogenesis of CAKUT. Evidence suggests that a large number of CAKUT are genetic in origin (see above) [4,5,12]. Maternal malnutrition, preeclampsia, diabetes, and congenital urinary tract obstruction can impair nephrogenesis during fetal life, resulting in a reduced nephron number at birth [15]. Many factors are also involved in the progression of kidney damage [16]. Albuminuria and arterial hypertension are not evident in all children with CAKUT but should regularly be assessed, as they are major factors that are associated with the progression of CKD and can be improved by therapeutic interventions [17]. Concerning the progression of kidney damage in CAKUT, two broad categories may be considered: those with a reduced number of nephrons (e.g., hypoplastic and dysplastic kidneys, solitary kidneys) and those with elevated hydrostatic pressure within the excretory apparatus (obstructive uropathies, vesicoureteral reflux, neurogenic bladder). In the first group, the kidney damage is congenital, and its progression is mainly due to glomerular hyperfiltration leading to glomerulosclerosis; in the second group, infections and decreased glomerular filtration, due to high intracapsular intrarenal pressure, are the main cause of progressive kidney damage. The outcomes for children with CAKUT are extremely variable in relation to the type of malformation and clinical characteristics of the single patient [17]. Oligohydramnios and impaired kidney function at birth are associated with a higher risk of ESRD in infancy as compared to children born after normal pregnancy with good postpartum kidney function [18]. A neonatal (after the first 48 h) serum cystatin C > 3.0 mg/L and serum creatinine > 0.6 mg/dL predicted progression to ESRD in a small group of newborns followed for an average of 6 years [19]. However, it has to be taken into account that kidney function tests in the first days of life are primarily based on maternal kidney function and cannot be used for the prediction of the development of CKD. The renal function is usually measured by the determination of the glomerular filtration rate (GFR), which does not represent the number of functioning nephrons since the “renal reserve” allows for an increase in the glomerular filtration, masking the loss of part of the parenchyma [16]. The progression of kidney damage in CAKUT is slower than in glomerular diseases [20] or hereditary diseases [21]. Therefore, in a substantial proportion of patients, RRT may be required in adulthood and not in pediatric age [22]. In a retrospective review of 113 children with PUV, Celakil and collaborators found that antenatal diagnosis and management of proteinuria and hypertension may modify this progression [23]. Hypertension has been detected in 19% of 333 CAKUT patients by Taha et al. and was more likely to be present in children with smaller kidneys, proteinuria, and reduced GFR. Hypertension increased the risk of developing CKD [24].

## 4. Kidney Transplantation

Kidney transplantation is the final goal for all children with ESRD and pre-emptive transplantation, especially by living donation, as it leads to the best graft and patient survival [25]. Between 30 and 50 percent of children who receive a kidney transplant (KTx) have CAKUT as an underlying disease [8]. Children and young adults with CAKUT were more likely to receive a kidney transplant compared to the others, and children with inherited causes of CAKUT have more chances to receive deceased transplants compared to those with anatomic causes of CAKUT [26]. Children with CAKUT who receive a KTx are usually younger and smaller and need more attention in the preparation for a kidney transplantation [27]. A selection bias may have influenced the results reported in the literature. Controlled prospective studies including detailed information on patients and procedures performed before, during, and after the transplantation will provide more reliable results in the future.

### 4.1. Pre-Transplant Investigations

Pre-transplant evaluation of CAKUT patients includes genetic, laboratory, and radiological investigations in addition to the standard preparation of non-CAKUT patients [28]. Preparation for kidney transplantation usually involves a multidisciplinary team to establish evaluation frameworks [7,19,29]. The International Children’s Continence Society (ICCS) has produced two consensuses: one on the evaluation and management of bladder bowel dysfunction [30] and another one on the therapeutic intervention in congenital neuropathic bladder and bowel dysfunction in children [31]; these recommendations should be followed accurately in children who will receive a transplant. Genetic studies are indicated in some cases of CAKUT and may provide reproductive counseling [32]. Laboratory examinations are usually performed on all children who enter a list of patients waiting for a kidney transplant [33]. The evaluation of kidney function is usually performed with the calculation of the glomerular filtration rate (GFR). The most used formula determines the estimated GFR from the serum levels of creatinine, cystatin C, and the height of the patient [34,35,36]. Glomerular hyperfiltration explains the finding of normal values of the glomerular filtration rate (GFR) in children with severe kidney damage and compensatory kidney growth in infants with a small single functioning kidney [37]. Renal functional reserve (RFR) is a measure of the increase of GFR in nephrons allowing the excretion of molecules maintaining their plasma concentration. In the early stages of CKD, the estimated GFR may remain within normal limits, but the renal reserve is diminished [38]. Unfortunately, no simple method has been developed to measure the RFR [39]. In children with lower urinary tract anomalies, a proper evaluation is important for renal transplant success [40]. Imaging investigations include: 1. Ultrasound (US). US provides an assessment of kidney shape and size and allows for the identification of hydronephrosis, kidney stones, or malignancy. Doppler US permits the identification of renal artery disease, renal vein thrombosis, and arteriovenous fistulas. US is relatively inexpensive but depends on the skill of the examiner. 2. Voiding cystourethrography (VCUG) allows for the evaluation of bladder morphology, voiding function, eventual urethral valves or obstruction, ureterocele or reflux and its grading; ultrasound cystography may be an alternative since it avoids radiation, but it provides less information; retrograde urethrography may be useful in cases of recurrent urinary tract infections or evidence of lower urinary pathology on kidney/bladder US [41]. 3. Nuclear Medicine. Dimercaptosuccinic acid (DMSA) radionuclide scan allows differential function of the two kidneys and detects kidney scarring; diethylenetriaminepentaacetic acid (DPTA) radionuclide scan and radionuclide scan with MAG-3 can reveal the presence of upper tract obstruction and are useful to rule out obstruction. Radionuclide cystography (RNC) reduces the radiation burden but does not permit evaluation of the bladder or the urethra. 4. Computed tomography (CT) provides a good resolution of the urinary tract. Sedation is often not necessary, but an IV contrast is needed in most cases. Radiation exposure is an important risk of CT, and its use should be limited to a small number of cases where the study is important for a correct diagnosis. In most children, US performed by an experienced examiner is sufficient for radiological evaluation. 5. Magnetic resonance studies (MRU) are also not usually recommended in children with CAKUT due to the need to sedate the child. MRU may be a suitable investigation in some cases, given the lack of radiation exposure. Functional magnetic resonance (FMR) urography and dynamic renal scintigraphy (DRS) are comparable in detecting urinary tract obstruction [42]. Urodynamic studies (UDS), including cystometry, electromyography of the urethral external sphincter muscle, urethrometry, and uroflowmetry, are mandatory in cases of lower urinary tract obstruction (LUTO), neuropathic bladder, ureterocele, and to assess bladder capacity, contractility, and emptying. A baseline urodynamic study, including VCUG, is indicated in all patients born with spina bifida within 3–12 months of life; in children with neurogenic lower urinary tract dysfunction, urodynamic testing should be performed annually until the child is 3 years old, and between the ages of 3 and 5 years old, only if upper tract changes or recurring UTIs are present; in children over the age of 5 years old, it should be performed when initiating a urinary continence program, or in the presence of hydronephrosis or renal scarring, recurring symptomatic UTIs, or changes in urinary continence status. Uroflowmetry may be sufficient in some cases [43]. Endoscopic evaluations are indicated when there is evidence of an anatomical problem. Renal biopsy is rarely needed in CAKUT.

### 4.2. Pre-Transplant Medical and Surgical Treatment

Medical treatment of children with CAKUT is not different from that of children with other kidney diseases [33]. Nutritional support, early introduction of growth hormone, and strict control of mineral bone disorder were helpful in reducing the progression of a small group of CAKUT patients followed from birth [19]. Treatment of hypertension is important to reduce the progression of kidney disease in CAKUT and renin–angiotensin–aldosterone system inhibitors (RAAS) are the first-line medication [44]. In a study of the German QiNKid (Quality in Nephrology for Children)-Registry children with CAKUT on dialysis required less intervention for hypoalbuminemia, fluid retention, anemia, hyperphosphatemia, and diastolic hypertension compared to those with glomerular diseases [45]. Vesico-amniotic shunt placement may be performed by experienced obstetric centers in selected cases pre-natally, but it is associated with a risk of fetal death, and there is no clear evidence in which cases it might improve kidney outcome [46]. Native nephrectomy may be indicated in a limited number of patients with CAKUT before transplantation if there is a history and therefore a risk of urinary tract infections (UTIs) [47]. Multicystic dysplastic kidneys should not be removed since they will involute in most cases, unless responsible for hypertension or recurrent UTIs. Ureterostomy provides temporary urinary diversion in children with complex urinary malformations and may therefore be indicated in some complicated cases. However, its risks, including UTIs, after kidney transplantation should be considered [48]. Vesicostomy performed before a kidney transplant in 16 children did not affect kidney function after 5 years despite UTIs [49]. Bladder augmentation may be considered in children with small capacity or low-compliant bladders [50]. Clean intermittent catheterization (CIC) is a simple procedure in girls, more cumbersome in boys, but older children may learn to do it. Vesicostomy is a simple, reversible procedure that decreases bladder pressure and UTIs and could be considered in children who do not reduce their bladder volume with catheter drainage; however, it may lead to incontinence as compared to CIC [51]. Augmented cystoplasty is a complex surgical procedure performed in children with small dysfunctional bladder refractory to other treatments and may be associated with complications [52]. Ureterocutaneostomy is rarely performed in recent days, but in a small number of patients, it may be the only option after kidney transplantation. A ureteroureterostomy of donor to native ureter may be performed if a post-transplant augmentation cystoplasty is planned. A major challenge is kidney transplantation in children with lower urinary tract disorders (LUTD), since anatomical and functional abnormalities may jeopardize the results of the procedure [53]. Pediatric patients with LUTD may have graft outcomes comparable with other pediatric renal transplant patients with careful preparation of the lower urinary tract [40]. A functioning bladder is essential for a successful transplant. The bladder should have low pressure and must empty completely and with no urinary leak. Management of neurogenic bladder (NGB) is a difficult task. Prompt imaging and urodynamic study followed by medical treatment and CIC are encouraged. A review of the medical management of NGB has been recently published and underlines the importance of a proactive approach including invasive interventions before the onset of renal abnormalities [54]. Behavioral, pharmacological, and surgical procedures should be accomplished by an active collaboration of pediatric nephrologists and urologists [55]. A detailed guideline on LUTD has been recently published [56] and others are available, but their applicability should be considered for future updates [57]. Children with functional LUTD may benefit from urotherapy according to indications of the International Children’s Continent Society (ICSS) Consensus [58]. Standard urotherapy includes patient education on regulating voiding patterns and avoiding constipation; specific urotherapy includes biofeedback therapy, alarms, and electrical neural stimulation. Pharmacotherapy of pediatric LUTD should be guided by the understanding of bladder activity. In the presence of an overactive bladder, the mainstay is anticholinergic therapy using agents that inhibit the binding of acetylcholine to the muscarinic receptors M2 and M3 on the detrusor smooth muscle cells; oxybutynin is the most used, while tolterodine, propiverine, and fesoterodine probably have less side effects. Alpha blockers (e.g., doxazosin) have a role in the management of children with dysfunctional voiding. Intravesical and intrasphincteric injection of botulinum toxin A may be an important therapeutic modality in children with neurogenic detrusor overactivity. Children with posterior urethral valves should have an endoscopic resection of the valves, but the bladder function does not always recover. In cases of high vesical pressure or residual postvoiding urine, CIC should be preferred to urinary diversion with vesicostomy or Mitrofanoff conduit and augmented cystoplasty [53]. A literature review on the management of valve bladder before a kidney transplantation has been published [59]. Vesico-ureteral reflux (VUR) is considered to be a risk factor for recurrent febrile urinary tract infections and renal transplant survival. In children with VUR, there is a significant variation in clinical practice among transplant surgeons. In a recent survey, almost 70% of surgeons from 40 pediatric KTx centers declared to treat symptomatic native kidney VUR before or during kidney transplantation [60]. An endoscopic intervention, instead of ureterocystoneostomy, is usually performed in most patients with VUR. In some patients with recurrent UTIs, native kidneys are nephrectomized at the time of Tx or shortly afterward to reduce the risk of infectious complications [61]. Unfortunately, almost all recommendations for surgical procedures before a kidney transplantation are based on retrospective not controlled studies or personal opinions; prospective controlled studies are needed.

## 5. Post-Transplant Management and Complications

Post-transplant complications in CAKUT patients are similar to non-CAKUT Tx recipients and include acute rejection, kidney hypoperfusion, viral infections, drug nephrotoxicity, and thrombotic microangiopathy. Obviously, there is no recurrence of the original disease in the Tx kidney. Urological complications are more frequent than in non-CAKUT patients and include urinary tract infections, urine leakage, vesico-ureteral reflux into the graft, graft urolithiasis, and urinary tract obstructions [62,63]. A survey on the surgical treatment is available in the literature [64].

Risk factors for UTIs in kidney transplanted adults are female gender, use of induction drugs, history of UTIs before KTx, acute rejection (AR), CMV infection, vesico-ureteral reflux or obstruction at the uretero-vesical junction, and especially persistence of CAKUT in the native kidneys as well as a neurogenic bladder after KTx [65,66]. UTIs are common in children post kidney transplantation and almost one-third have pyelonephritis [67]. Post-transplant febrile UTIs are more frequent in children with CAKUT compared with patients without; a temporary decrease of graft function during the infection has been reported [8,68]. UTIs are not associated with increased graft loss or mortality if early detected and properly treated but may reduce graft survival if frequent and not treated early enough [69]. A multicenter prospective study confirms a high incidence of febrile UTI (fUTI) after pediatric KTx, which affects graft function during the infectious episode but not after two years [68]. UTIs may be related to the transplant procedure and medications [70]. Yazici et al. evaluated 52 adults with reflux nephropathy compared with 42 patients with glomerular disease followed for six years after kidney transplantation; a higher incidence of UTI was observed, while acute rejection, graft failure, and renal function were comparable [71]. A similar study in 31 adults with reflux nephropathy compared with 31 patients with chronic glomerulonephritis showed a higher incidence of UTI; nephrectomy before kidney transplantation did not change the rate of complications and is rarely indicated [72]. Kamal et al. compared 20 children with posterior urethral valves (PUV) to the other 277 who received a kidney transplant and found more UTI, urinary fistula, and acute rejection in the first group [73]. In a retrospective analysis of 45 patients with a diagnosis of PUV who underwent kidney transplant, a higher rate of UTIs was noticed but no significant difference in graft function outcomes was observed in those who received bladder management consisting of frequent voiding, CIC, and diversion augmentation cystoplasty [74]. Patients who undergo bladder augmentation have a higher risk of UTIs [75]. Kidney transplantation in pediatric patients with vesicostomy seems to be a safe and effective strategy. The UTI rate in pediatric patients undergoing kidney transplantation with vesicostomy may be similar to that reported in patients without urinary diversion [49]. Costigan et al. did not find an increased occurrence of UTIs in kidney-transplanted children with ureterostomy [48]. An increase in pyelonephritis despite antibiotic prophylaxis has been reported in nine children who had diagnoses of VACTERL association [76]. Good hydration, frequent voiding, antimicrobial prophylaxis, and removal of ureteral stents may reduce the incidence of UTIs [77]. Hospitalizations for UTIs in pediatric kidney transplant recipients with CAKUT are not different from the others with no CAKUT [70].

Vesico-ureteric reflux (VUR) into transplanted kidneys in children is a common occurrence and can also be detected in children who had an anti-reflux procedure. Transplant pyelonephritis may produce irreversible damage to the graft, which may be avoided with a prompt antibiotic treatment [78]. A European survey has revealed that most centers correct the reflux in native kidneys in selected patients preferably before the KTx with an endoscopic treatment. Almost all perform an anti-reflux ureteral reimplantation using an extravesical technique, but management for symptomatic allograft VUR varies, implying a need for prospective studies [61]. In some cases, VUR of the transplanted kidney has to be treated by endoscopic or surgical anti-reflux procedures. Ambulatory blood pressure monitoring for 24 h (ABPM) in kidney transplanted children has shown that, as in adults, nocturnal hypertension or a blunted nocturnal dipping are frequent. It is not known if these abnormalities may affect the graft function [79]. Urodynamic investigations should be repeated after a kidney transplant as part of a surveillance regimen for patients at high risk of urinary tract complications [56].

## 6. Long-Term Outcome

Several investigators have analyzed the long-term outcome of kidney transplantation in patients with CAKUT. All studies are retrospective, and patients’ selection, kind of disease, pattern and duration of dialysis, year of transplant, donor features, mismatch rates, procedures performed before or during surgery, medications received, and time of follow-up affect the results as in all patients after pediatric KTx. Most studies have shown comparable graft and patient survival in children with CAKUT [29]. Haberal et al. have recently reviewed the literature and selected 15 studies which show that kidney graft survival up to 10 years is not different in children with LUTD from the others. Higher graft survival has been reported in the VUR nephropathy group, while patients with bladder dysfunction have a decline in graft function over the long term. UTIs are more frequent in patients with LUTD [80]. Old data showed a low rate of graft survival in patients with CAKUT. Crowe et al. reported a graft loss at 5 years in 55 adult patients with CAKUT and normal or abnormal bladders of 75% and 57%, respectively [81]. McKay et al. extracted from the Australian & New Zealand Dialysis & Transplant Registry the outcomes of kidney transplant in 127 patients with posterior urethral valves (PUV), 727 with reflux nephropathy and 245 with renal hypoplasia/dysplasia. A 10-year graft survival in PUV, hypoplasia/dysplasia, and reflux nephropathy was 70%, 76%, and 70%; recipients with PUV had an increased risk of graft loss beyond 10 years post-transplant [82]. Cornwell et al. examined 10,635 first-time renal transplant patients (adults and children) with CAKUT transplanted between 1995 and 2018. They compared graft survival between CAKUT vs. non-CAKUT and examined those with lower urinary tract malformations (LUTM) vs. upper urinary tract malformations (UUTM). Graft survival was better in CAKUT patients with a 5-year survival; comparison of LUTM to UUTM showed no difference; examining pediatric LUTM alone, graft survival was not better than matched non-CAKUT [83]. Oto et al. compared 169 adults with CAKUT and 169 matched controls in Istanbul and found that graft survival rates at 10 years were significantly higher in the CAKUT group than in the non-CAKUT group. Patients in the CAKUT group were younger, and the most common disease was VUR nephropathy (76%) [84]. Monteverde et al. examined 923 transplanted children with CAKUT: graft survival, up to 10 years, was better in CAKUT recipients vs. those with primary glomerular diseases; the main predictors of graft loss were delayed graft function, late acute rejection, and age at KTx in the CAKUT group [8]. Similar results have been reported in other studies with a limited number of patients [40,69,73,76,85,86,87]. Hussein et al., in a group of 123 transplanted children, found that those with urological causes of CKD had comparable graft survival to the others after almost 4 years of observation but had reduced renal function and more complications; almost half of their patients had PUV [88]. Similar results have been reported by Adams et al. in a retrospective review of 66 children with malformations of the lower urinary tract. At 5 years, the graft survival rate was 70% for all children, 78.5% for children with VUR, 62.9% for PVU, and 50% for neurogenic bladder [89]. Silverii et al. followed for a median of 5.4 years 45 patients with PUV who received different bladder management including CIC, diversion, and augmentation cystoplasty; patients on CIC had higher rates of UTI, but no significant difference in graft function outcomes [74]. Kaya Aksoy et al. examined 24 children with vesicoureteral reflux nephropathy, 25 with posterior urethral valve, and 9 with neurogenic bladder. The 5-year graft survival rates of patients without lower urinary tract dysfunction and those with vesicoureteral reflux nephropathy were similar (51.3% vs. 51.6%) [90]. Marchal et al. noticed that graft survival at 10 years was 77.3% in a retrospective multicenter study from French renal transplant centers on 112 patients with LUTM; the survival was similar to other kidney transplantations on native bladders. Pyelonephritis was more frequent in patients who received enterocystoplasty or continent urinary diversions [91]. Wilson et al. compared three groups of transplanted patients: 70 with polycystic kidney disease, 16 patients with LUTD with prior intervention, and 64 with LUTD without intervention end-stage renal disease. Five-year patient survival was 100%, 100%, and 94.3%, respectively [92]. Patry et al. analyzed 62 children from the CERTAIN Registry and found that there was no difference in 5-year graft survival comparing patients with pre- or post-transplant surgical reconstruction of the lower urinary tract. They also noticed that transplant dysfunction was more frequent in children with LUTD than in controls [93].

## 7. Future Directions

A large number of scientific papers are available in the literature dealing with the medical and surgical management of patients with CAKUT before, during, and after a kidney transplant. The pre-transplant medical and surgical procedures are different among centers, complications described vary, and long-term results seem comparable to kidney transplantation performed in patients with different kidney diseases. All studies are retrospective, and the selection of patients admitted to a transplant influences the results. In our review, we summarize recent publications and highlight potential future research directions in kidney transplantation in CAKUT. New technologies are available, and new prospective studies could be organized. To harmonize the work-up and treatment of children with CAKUT, scientific societies should promote systematic reviews and evidence-based guidelines.

## Figures and Tables

**Table 1:** Classification of congenital anomalies of the kidney and urinary tract (CAKUT).

**Kidney Anomalies:**
Renal agenesis
Renal hypoplasia
Renal dysplasia
Renal malrotation
Horseshoe kidney
Dystopic kidney
**Upper urinary tract:**
Ureteropelvic junction obstruction
Megaureter
Duplex collecting system
Hydronephrosis
**Lower urinary tract:**
Urethral valves
Vesicoureteral reflux
Bladder diverticula
Urethral stenosis
Hypospadias
Ureterocele
Neurogenic bladder
**Syndromic CAKUT:**
Prune-belly syndrome
VATER syndrome
VACTRL syndrome

N. B.: In many cases, a combination of some or multiple anomalies can be found.

## Data Availability

Data are contained within the article.

## Abbreviations

Urinary tract infection	(UTI)
Febrile UTI	(fUTI)
Congenital anomalies of the kidney and urinary tract	(CAKUT)
Kidney transplant	(KTx)
Renal replacement therapy	(RRT)
Voiding cystourethrography	(VCUG)
Chronic kidney disease	(CKD)
Glomerular filtration rate	(GFR)
Lower urinary tract disorders	(LUTD)
Lower urinary tract malformations	(LUTM)
Clean intermittent catheterization	(CIC)
Neurogenic bladder	(NGB)
Vesicoureteral reflux	(VUR)
Renal functional reserve	(RFR)
Ultrasound	(US)
Dimercaptosuccinic acid	(DMSA)
Diethylenetriaminepentaacetic acid	(DPTA)
Computed tomography	(CT)
Voiding cystourethrography	(VCUG)
Magnetic resonance studies	(MRU)
Posterior urethral valves	(PUV)
Renin angiotensin aldosterone system inhibitors	(RAAS)
Lower urinary tract obstruction	(LUTO)
Functional magnetic resonance	(FMR)
Dynamic renal scintigraphy	(DRS)
Urodynamic studies	(UDS)
